# The Effectiveness of High Intensity Laser in Improving Motor Deficits in Patients with Lumbar Disc Herniation

**DOI:** 10.3390/life14101302

**Published:** 2024-10-14

**Authors:** Diana-Lidia Tache-Codreanu, Magdalena Rodica Trăistaru

**Affiliations:** 1 Neurorehabilitation Research Laboratory, Medical Rehabilitation Department, Colentina Clinical Hospital, Stefan cel Mare Street No. 19–21, 020125 Bucharest, Romania; 2 Department of Physiotherapy, University of Medicine and Pharmacy, Petru Rares Street No. 2, 200349 Craiova-Dolj, Romania; rodicatraistru@hotmail.com

**Keywords:** assessment, disability, high intensity laser therapy, motor deficit, disk herniation, neuronal recovery, rehabilitation program

## Abstract

Background: High-Intensity Laser (HIL) therapy, known for its biostimulatory effects on nerve cell growth and repair, shows promise for improving motor deficits caused by morphopathological changes. This research study aimed to comprehensively assess muscle strength changes through muscle testing, complemented by functional tests evaluating factors contributing to disability in patients with Lumbar Disc Herniation (LDH) and associated motor impairment, following a complex rehabilitation protocol incorporating HIL therapy. Methods: A total of 133 individuals with LDH and motor deficits were divided into two groups. Group 1 (*n* = 66) received HIL therapy followed by standard rehabilitation, while Group 2 (*n* = 67) underwent only the standard rehabilitation program. Functional parameters, including muscle strength, the ability to walk on tiptoes or heels, and self-assessed fall risk, were monitored. Results: Both groups showed statistically significant improvements in all monitored parameters. A comparative analysis revealed a significant result for the HIL therapy regimen across all indicators. Conclusions: The group undergoing a rehabilitation program with integrated HIL therapy displayed significantly greater improvement in motor deficits, affirming the positive impact of HIL therapy on functional parameters among LDH patients.

## 1. Introduction

LDH resulting in nerve root compression stands as the leading cause of lumboradicular pain, prevailing in approximately 90% of cases [1]. The pathophysiology of LDH with radicular involvement encompasses morphopathological alterations in the intervertebral disk, vertebra, and adjacent radicular nervous structures. Paresis and other neurological motor symptoms are commonly observed, occurring in 30–50% of symptomatic patients with disk herniation [2]. The treatment approach for LDH with motor deficits typically involves specific pharmaceutical interventions, physical therapies, kinesiotherapy, orthotic devices, and, as a last resort option, lumbar decompressive surgery. However, while the effectiveness of such interventions is undeniable, not all patients may be suitable candidates, and the recurrence of symptoms remains between 3% and 43% of patients [3]. The manifestation of motor deficits in LDH can be viewed as a complication and a setback in the management of lumbo-radicular pain, contributing significantly to the escalating global costs associated with lumbosciatica, including work-absence and impairment in socio-familial activities. Hence, the development of an efficacious, comprehensive rehabilitation program is desirable [4,5,6].

Lasers, particularly Low-Level Laser (LLL) and High-Intensity Laser (HIL), are commonly utilized in rehabilitation medicine. The therapeutic effects of LLL in mitigating pain, inflammation, edema, and promoting nerve healing have been recognized for nearly four decades [7,8]. Compared to LLL, HIL sources possess superior penetration capabilities, with their effects amplifying with increasing dose and power [9]. Considering the fact that a laser source with a wavelength of 1064 nm was used, it can be assumed that the penetration depth reaches at least 50 mm, stated for the range of 630–1100 nm for LLT [10].

The mechanism of action for HIL therapy encompasses physiological, anti-inflammatory, thermal, and clinical effects. Physiological effects involve enhanced oxygen supply, increased activity of intracellular enzymes, DNA synthesis stimulation, Na/K membrane pump activation fostering metabolic processes, and modulation of local histamine, prostaglandin, and endorphin levels. The anti-inflammatory effect is mediated by immune system cell stimulation, reducing prostaglandin levels (PGE2), and promoting prostacyclin synthesis (PGI2) [9,11,12]. HIL therapy facilitates nerve structure restoration via Schwann cell stimulation [13]. The thermal effect induces muscle relaxation and analgesia in trigger points. Clinically, HIL therapy yields biostimulatory, anti-inflammatory, anti-edematous, vasodilating, and pain-reducing outcomes [9,14,15].

Although the effects of HIL therapy are widely recognized and comprehensively documented, its specific impact on motor deficits arising from LDH lacks adequate clinical support. The impact of laser energy on neurological tissue has been extensively studied, particularly in animal models such as rats. Michel et al. demonstrated a notable effect of laser therapy in preventing the reduction in myelin sheath thickness and halting myelin degeneration through methods including LFB staining and myelin basic protein (MBP) immunohistochemistry [16]. Similarly, Alcãntara et al. observed a favorable impact on the upregulation of the axonal growth marker, specifically MMP-9, and the modulation of TNF-α protein levels during the acute phase of nerve injury [17]. While acknowledging the experimental nature of these animal studies, their findings underscore the substantive evidence supporting the beneficial effects and therapeutic potential of laser therapy in nerve tissue stimulation. A comprehensive review of clinical literature focusing on human subjects has corroborated the efficacy of HIL therapy, either as a standalone intervention or in combination with adjunctive modalities, in mitigating back pain and associated disability, within the cervical or lumbar regions [18,19,20,21,22,23,24,25,26,27,28]. A minority of investigations did not demonstrate positive outcomes and deemed HIL therapy ineffective for addressing LDH [29,30]. Notably, most studies focusing on patients with back problems primarily evaluate pain outcomes, with some also assessing functional parameters such as range of motion and subjective disability assessment. While these parameters are closely intertwined with pain, and likely improved due to pain reduction, there remains a paucity of research directly evaluating the effect of HIL therapy on motor deficits and their symptomatic manifestations.

The main goal of the study is to ascertain the effectiveness of a novel rehabilitation strategy targeting the peripheral nervous system in alleviating motor deficits stemming from LDH by incorporating HIL therapy followed by conventional rehabilitation, along with methods to evaluate functional walking disorders and the self-assessed risk of falls due to motor deficits, both of which contribute to disability in these patients. Outcomes will undergo comprehensive analyses and be compared with results observed following conventional rehabilitation alone.

## 2. Materials and Methods

A total cohort of 133 patients (84 females and 49 males) underwent a treatment regimen at the Rehabilitation Department, Neurorehabilitation Research Laboratory, Colentina Clinical Hospital in Bucharest, Romania, spanning the period between 2012 and 2022. Throughout the study duration, no adverse effects were noted following the application of HIL therapy.

The participants were randomized into two groups: Group 1 comprised 66 individuals (44 females and 22 males), while Group 2 consisted of 67 individuals (40 females and 27 males). The patients were subjected to a random assignment and divided into two groups: Group 1 underwent the standard rehabilitation protocol followed by HIL therapy, while Group 2 received the standard rehabilitation protocol solely. Each treatment regimen spanned a duration of 10 days. Randomization was executed via a computer program in blocks of 10 patients. The principal physiotherapist managed patient enrollment and group assignment, as well as administered the HIL therapy treatment. Another clinician, uninformed of group allocation, provided the standard rehabilitation protocol for both groups.

Patients were diagnosed with a paretic motor deficit arising from LDH at the L4–5 or L5–S1 level, exhibiting a minimum muscle strength of −3 in the affected muscle (either the tibialis anterior muscle or triceps surae muscle) and classified as Fitzpatrick skin type IV, were considered eligible for inclusion in the study if they were within 6 ± 1 months from the onset of the neurological deficit [9,31]. The 6 month timeframe was selected based on the likelihood of favorable outcomes following surgical treatment of LDH without a progressive motor deficit [32]. It has been established that those neurological deficits persisting beyond the 6 month period do not detrimentally affect surgical outcomes; hence, patients with prolonged deficits could potentially still benefit from invasive procedures if conservative approaches fail [33,34].

The exclusion criteria encompassed patients with surgical indications for a motor deficit due to LDH, dermatological disorders, fever, neoplastic diseases, decompensated chronic illnesses, as well as pregnant and menstruating women. Additionally, individuals exhibiting hypoesthesia in the lumbar region, cochlear implants, metallic implants, undergoing treatment with photosensitive drugs, or classified as having high skin type (Fitzpatrick skin types V or VI) were excluded. All participants underwent neurosurgical or spinal surgery examinations to identify indications for surgery, such as progressive and significant lower limb weakness indicative of myelopathy or cauda equina syndrome. Only patients lacking absolute indications for surgery and thus eligible for conservative treatment were enrolled [35].

Given the exploratory nature of this pilot study, the determination of the sample size was not predicated on prior research findings [36].

The standard rehabilitation program comprised physical therapy (including, in order, device therapy, therapeutic massage and exercises), orthotic therapy (utilizing lumbosacral orthosis, supplemented by foot and ankle orthosis if deemed necessary), and pharmacotherapy (consisting of anti-inflammatory, myorelaxant, and analgesic medications). All patients followed the same treatment. The device therapy encompassed the following succession of modalities:

1. Thirty-minute sessions of low-intensity magnetic field application to the lumbar region (comprising a series of magnetic rectangular pulses with a pulse frequency of 3 Hz and an intensity of 38.0 mT) and to the limb exhibiting paretic muscle (comprising a series of magnetic rectangular pulses with a pulse frequency of 16 Hz and an intensity of 261.0 mT), intended to stimulate tissue regeneration and elicit anti-inflammatory and analgesic effects.
2. Thirty-minute sessions of immersion in four galvanic cell baths, targeting enhanced muscle and nerve excitability and vasodilatory effects.
3. Electrostimulation employing exponential monophasic impulses tailored to the triceps surae or tibialis anterior muscle, with an individualized intensity/time curve aimed at mitigating muscle atrophy.
4. Lower limb whirlpool baths to augment oxygen supply to muscle fibers via heightened blood circulation.

HIL therapy was exclusively administered to Group 1, and it was applied at the beginning of the therapeutic program, comprising 10 daily sessions, targeting the bilateral paravertebral lumbar region, with a treatment area of 25 cm^2^ and a wavelength of 1064 nm. The energy was delivered in two successive phases. Phase 1 aimed at achieving analgesic effects, utilizing pulsed mode at a frequency of 25 Hz, with circular movements applied for 1 min and 40 s, a power of 10 W, and a dose of 10 J/cm^2^. Phase 2 focused on bio-stimulation effects, employing continuous mode with continuous movements for 6 min and 56 s, a power of 6 W, and a dose of 100 J/cm^2^. The protocol was devised in accordance with the Arndt–Schultz rule, which delineates the dose-dependent effects in laser therapy, expressed as $DE=\frac{P\cdot x\cdot t}{A}$; where *DE* represents energy density (J/cm^2^), *P* is power (W), *t* is exposure time (s), and *A* is the laser focus area (cm^2^) [14]. Notably, the sensation induced on the area treated by HIL therapy was characterized by a slight warming of the skin.

To comprehensively analyze the progression of motor deficits attributable to LDH, three functional parameters were assessed: muscle strength, the ability to walk on tiptoes or heels, and the risk of falls stemming from motor impairment. These outcome measures were evaluated both prior to the commencement of the treatment program and immediately upon its completion.

Muscle strength was evaluated using the National Foundation for Infantile Paralysis Numeric Scale (1946), as further refined by L. Daniels and C. Warthingham [37]. This assessment involves evaluating the strength of muscles against varying resistance intensities or their ability to move against gravity, with or without gravitational assistance, and the presence of muscle twitching or movement. For the statistical analyses, the results were transformed utilizing the 12-point scale described by Kendall FP (1993) (Table 1) [37].

Another parameter employed was the subject’s ability to walk on their tiptoes or heels. LDH at the L5–S1 level notably restricts walking on tiptoes, while LDH at the L4–L5 level diminishes the ability to walk on heels. A 4-point scale was employed for this assessment: 0—feasible without limitations, 1—feasible with some difficulty, 2—feasible but with significant difficulty (inability to sustain dorsiflexion of the foot throughout the support phase of walking), 3—unfeasible.

The risk of falls attributed to motor deficit was evaluated using a self-assessed 3-point scale: 0—no risk of falling, 1—low risk of falling, and 2—high risk of falling.

Demographic details (age, gender, occupation, physical workload, and body mass index (BMI)), as well as the level of herniated disk (L4–L5 or L5–S1) and surgical history of patients, were recorded for comparison of distribution between the two groups. The influence of surgical history on therapy outcomes was further scrutinized for both groups.

Statistical analyses and data processing were conducted using SPSS 23 software. The measured variables were represented using charts and tables. Given the relatively small-scale nature of the data, which did not adhere to a normal distribution according to the Shapiro–Wilk test, non-parametric tests were employed to ascertain statistical significance. The only parameter that showed a normal data distribution was the age of the subjects; therefore, the distribution of patients into groups according to this parameter was evaluated by the two sample *t*-test, while the distribution according to BMI was evaluated by the non-parametric Mann–Whitney U test. The Chi-square test was used to analyze participant distribution concerning categorical data (gender, lesion level, surgical status and physical load) across both groups. Additionally, the Chi-square test was used to assess the impact of surgical status on the degree of improvement in muscle strength and risk of falls parameters. As the test deals with categorical data, continuous outcomes were transformed into categorical forms, facilitating the statistical analyses and data interpretation. The Wilcoxon signed-rank test was used to compare motor deficit indicators obtained before and after the completion of the treatment program, while the Mann–Whitney U test was applied to compare the extent of improvement achieved between the two groups. A significance level of *p* < 0.05 was deemed statistically significant.

## 3. Results

The distribution of patients across demographic and other parameters into these groups is delineated in Table 2. The majority of participants were within the age range of 40 to 69 years, with no participants below the age of 30. Notably, no significant between-group disparities were observed in terms of age, BMI, or gender distributions.

### 3.1. Muscle Strength

**Figure 1:**
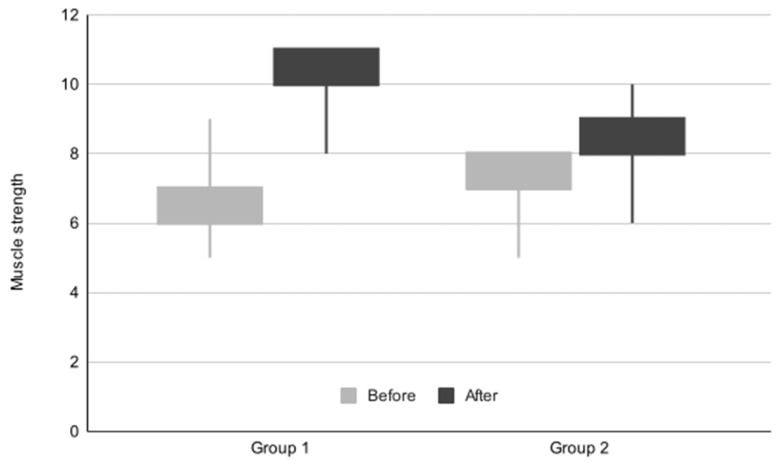
Box plot graph representing evolution of muscle strength scores for Group 1 and Group 2.

The initial and final median scores of muscle strength are delineated in Table 3. Notably, both patient groups exhibited statistically significant enhancements in muscle strength throughout the treatment regimen. Although the initial muscle strength values were identical for both groups (as indicated in Table 3), the Mann–Whitney U test identified them as statistically significantly different in favor of Group 2. Specifically, patients in Group 2 demonstrated a higher average muscle strength prior to commencing the treatment program (6.99 ± 0.84) compared to those in Group 1 (6.56 ± 1.04). However, the improvement observed during treatment was more pronounced in patients from Group 1, as confirmed by the statistically significant between-group comparison utilizing the Mann–Whitney U test. The progression of the muscle strength score throughout the treatment program for both groups is depicted in Figure 1.

The impact of the surgical status of LDH on the muscle strength score was assessed, revealing no significant difference within the entire study population (χ^2^ = 7.045, 10 degrees of freedom, *p* = 0.721) or within individual groups (χ^2^ = 6.803, 10 degrees of freedom, *p* = 0.744 for group 1; χ^2^ = 5.606, 10 degrees of freedom, *p* = 0.847 for group 2). The disparity between muscle strength scores before and after treatment, and contingent upon the surgical status, is detailed in Table 4.

### 3.2. The Ability to Walk on Tiptoes or Heels

**Figure 2:**
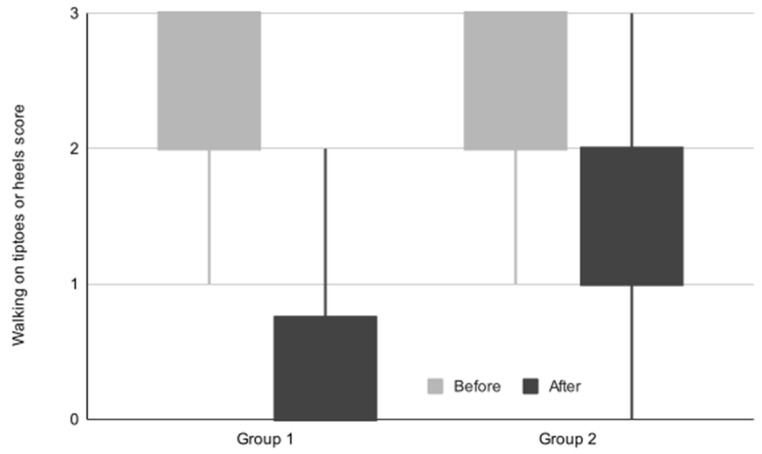
The evolution of the ability to walk on tiptoes or heels score.

The ability to walk on tiptoes or heels score obtained before and after the treatment program for both groups is displayed in Table 5 and Figure 2.

Throughout the treatment program, both cohorts exhibited statistically significant improvements in their ability to walk on tiptoes or heels, with Group 1 showing a median increase of 2 points and Group 2 showing a median increase of 1 point. Notably, 74.24% of participants in Group 1 and 16.42% in Group 2 attained a final score of 0, indicating substantial progress. The observed outcome favored Group 1 significantly, as confirmed by rigorous statistical analysis employing the Mann–Whitney U test for between-group comparison.

### 3.3. Risk of Falls

**Figure 3:**
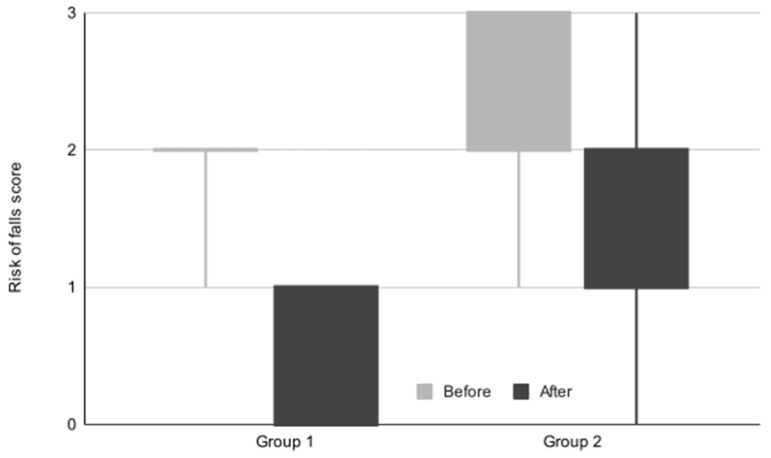
The evolution of risk of falls score.

Table 6 displays the self-assessed risk of falls scores for both groups. Throughout the treatment program, both cohorts experienced statistically significant improvements in this parameter. A statistical between-group comparison regarding the risk of falls parameter indicated a significant difference between the values obtained before and after completing the treatment program. The difference in the after values was more pronounced, as inferred from the median values for individual groups. The evolution of the risk of falls score is illustrated in Figure 3.

The surgical status exerted no significant influence on the progression of the risk of falls parameter in either of the study groups (χ^2^ = 0.156, 4 degrees of freedom, *p* = 0.997 for group 1; χ^2^ = 5.089, 4 degrees of freedom, *p* = 0.278 for group 2) or for the entire study population (χ^2^ = 1.558, 4 degrees of freedom, *p* = 0.816). The disparity in scores obtained before and after the treatment program based on surgical status is detailed in Table 7.

The study participants exhibited lesion levels at two possible locations: lumbar, defined as lesions at the L4–L5 vertebral level, and sacral, defined as lesions at the L5–S1 vertebral level. The analyses of patient distribution across individual groups based on lesion level did not yield a statistically significant between-group difference. The majority of enrolled participants (58.7%) presented with herniations in the L4–L5 area, while 41.3% had herniations in the L5–S1 region.

The surgical status of LDH was deemed a pertinent factor potentially impacting treatment outcomes (see Table 2). Subjects were categorized into three groups: not operated (NS—no surgery), operated (S—surgery), or iteratively operated (IS—iterative surgery). However, no statistically significant between-group difference was evident. Patients who had undergone surgery completed the treatment program within 3 to 12 months post-intervention. Since all patients were within 6 ± 1 months from the onset of neurological deficits, the time elapsed since surgery was not factored into subsequent analyses.

Considering patients’ occupation and physical activity levels, a classification into five categories was conducted: sedentary (S), medium physical activity (M), intense professional physical activity with overload (IP), intense physical activity with extended fixed position (IF), and intense physical activity in the household (IG). Notably, there was a statistically significant difference in patient distribution across these groups in terms of physical load. Specifically, patients in Group 2 exhibited slightly higher physical activity levels compared to those in Group 1.

## 4. Discussion

The current study aimed to assess the efficacy of a novel rehabilitation strategy targeting the peripheral nervous system, incorporating conventional rehabilitation with HIL therapy, in ameliorating neurological motor symptoms among patients with motor deficits attributed to LDH. The assessment focused on changes in muscle force through muscle strength tests and two functional tests evaluating walking disorders causing disability due to motor deficits: the ability to walk on heels or tiptoes, and self-assessed risk of falls. These parameters are infrequently evaluated in existing clinical evidence concerning non-invasive LDH treatment, which predominantly focuses on pain and disability manifestations rather than neurological deficits. Thus, this study specifically targeted patients experiencing motor deficits and aimed to evaluate the reduction in their symptoms through HIL therapy.

The findings revealed significantly superior outcomes among participants receiving HIL therapy in conjunction with conventional rehabilitation compared to those receiving stand-alone conventional therapy for motor deficit rehabilitation due to LDH. This disparity reached statistical significance across all monitored parameters (muscle strength, ability to walk on tiptoes or heels, and risk of falls attributed to motor deficit). The study’s hypothesis, which posited a positive impact of HIL therapy on motor deficit improvement in LDH with no observed side effects, was thus confirmed.

As this study represents a pilot investigation with a distinctive focus, a direct quantitative comparison of its findings and conclusions with existing clinical evidence is challenging. Nonetheless, the observed positive impact of HIL therapy on lower back conditions aligns with conclusions drawn from prior research [21,22,23,24,25,26,27,28]. Systematic reviews and meta-analyses conducted by Abdildin et al. and Alayat et al. have documented the efficacy of HIL therapy in mitigating LDH symptoms, including pain and functional limitations [21,22]. Moreover, Fiore et al. and Boyraz et al. have explored the comparative effectiveness of HIL therapy versus ultrasound treatment in individuals with low back pain [23,26]. Fiore et al. reported HIL therapy to be more efficacious in reducing pain and disability, while Boyraz et al. observed comparable effects of HIL and ultrasound therapy on mental health and enduring pain relief, in contrast to conventional exercise. Abdelbasset et al. demonstrated the superiority of HIL therapy over pulsed electromagnetic field therapy in alleviating pain, reducing disability, and improving range of motion among patients with non-specific chronic low back pain [24]. Similarly, Alayat et al. found HIL therapy combined with exercise to be more effective than HIL alone or placebo with exercise in addressing pain, disability, and range of motion in chronic low back pain patients [25]. Chen et al. compared the effectiveness of a combination of HIL and spinal decompression system versus a stand-alone spinal decompression system, revealing that HIL therapy contributed to enhanced disability and range of motion, while pain levels remained consistent across both groups [27]. Furthermore, Tramontana et al. delved into the biostimulating effects of laser therapy, uncovering a statistically significant increase in mucopolysaccharides and newly formed elastic fibers among patients undergoing HIL stimulation compared to those receiving placebo treatment, as evidenced by their histological analysis [28]. Despite the compelling evidence supporting the potential benefits of HIL therapy for lower back conditions, the existing literature predominantly overlooks neurological manifestations associated with disk herniation.

The majority of studies investigating non-invasive methods for LDH overlook neurological motor deficits, making quantitative comparisons with the outcome measures of the present study challenging. A single case study stands out, demonstrating improvement in muscle strength and walking ability in a patient with LDH following a non-invasive rehabilitation program centered on physical exercise and stretching [38]. However, due to its limited sample size and modest improvement, direct quantitative comparison is impractical. Notably, microdiscectomy for LDH has shown significant improvement in muscle strength, with up to 75% of patients achieving complete recovery [39]. Although direct comparison of results is hindered by differences in quantification methods, the surgical focus on alleviating compression suggests potentially more pronounced outcomes post-surgery compared to non-invasive interventions.

A secondary observation of this study is the efficacy of HIL therapy independent of the surgical status of LDH patients (non-operated, operated, and iteratively operated). This indicates promising results of HIL therapy not only in non-operated individuals but also in those who have undergone surgery yet still experience persistent or recurrent motor deficits. This finding resonates with research by Erdem et al., which underscored the efficacy of non-invasive treatment programs for patients experiencing recurrent symptoms post-lumbar discectomy [40].

### 4.1. Limitations of the Study

It is essential to acknowledge several limitations in this study. One notable limitation is the absence of a follow-up period, which would enable the assessment of long-term therapy effectiveness. Additionally, patient stratification into study groups, based on demographic and other indicators such as age, gender, BMI, lesion level, surgical status, and physical load, was lacking. While most parameters did not exhibit significant between-group differences, uneven distribution regarding physical load favored patients in Group 1, potentially influencing therapy outcomes [41]. Moreover, the absence of sham laser treatment for the control group could be considered another limitation. Although the intensive conservative treatment program might have led to improvement in the control group, non-blinding of the patients could have affected the results, suggesting a need for methodological refinement in future studies. A thorough assessment using the Cochrane risk-of-bias tool for randomized trials (RoB 2) was conducted to gauge the potential biases present [42]. The randomization process and measurement of the outcome domains raised “some concerns” due to the lack of stratification of treatment groups and the absence of sham laser treatment in Group 2, potentially leading to subjects’ awareness of their group assignment. However, the remaining three domains were deemed to have a “low” risk of bias, despite minor shortcomings in the study design. It is imperative to acknowledge this potential bias and interpret the results cautiously until corroborated by further research with meticulously crafted protocols.

### 4.2. Future Developments

Considering study limitations, further research could benefit from a longer follow-up period and the incorporation of objective quantitative methods like electroneurography. Analytical evaluation of lesions amenable to regeneration through HIL therapy could provide a more precise assessment of its therapeutic effect on neuronal recovery. The results obtained from this study, along with the known mechanism of action of HIL therapy, encourage further investigation into its efficacy within the context of Enhanced Recovery After Surgery (ERAS) protocols for spine surgery [43]. Integrating HIL therapy into postoperative care regimens has the potential to mitigate complications and pain, leading to a decreased reliance on analgesics, improved healing and tissue regeneration, and overall hastened recovery. Comparing recovery metrics between patients undergoing standard postoperative care and those receiving HIL therapy could shed light on its impact on disability and hospital stay duration, offering valuable insights into its role in optimizing post-surgical recovery in lumbar spine surgery patients. Implementing propensity score methods in future research endeavors could address the limitations observed in the current study, such as the lack of group stratification and inadequate randomization. By leveraging these methods, researchers can effectively estimate treatment effects while accounting for existing confounding factors, thus enhancing the validity and reliability of study findings. Indeed, leveraging propensity score methods in future research endeavors could address the limitations observed in the current study. By employing these methods, researchers can effectively estimate the treatment effect while accounting for existing confounding factors, thereby enhancing the validity and reliability of study findings. This approach offers a more efficient and cost-effective alternative to creating artificial control groups, allowing for comparisons with existing evidence obtained through conventional methods or surgical approaches. It is essential to systematically monitor and account for potential confounding factors within the target patient population across studies, while also striving to standardize evaluation methodologies to facilitate meaningful comparisons and advancements in the field.

## 5. Conclusions

While both patient groups demonstrated statistically significant improvements in all monitored parameters, the comparison between groups highlighted a significant difference favoring the HIL therapy supplemented program across all indicators. These findings imply a beneficial influence of HIL therapy on functional parameters in LDH patients. However, to solidify these conclusions, further research is warranted, particularly focusing on long-term effectiveness, and complementing it with objective quantitative methods like electroneurography. The study encourages further research to validate and potentially extend the potential of HIL therapy, particularly in the context of postoperative recovery, thus contributing to advancements in clinical practice and patient care.

## Figures and Tables

**Table 1:** The 12-point scale described by Kendall F.P. (1993).

Grade	Description	Muscle Strength Score	Points
No movement	Holds test position against strong pressure	5	12
	Holds test position against moderate to strong pressure	4+	11
Supported in Horizontal Plane	Holds test position against moderate pressure	4	10
	Holds test position against slight to moderate pressure	4−	9
	Holds test position against slight pressure	3+	8
Tests in Antigravity Position	Holds test position (No added pressure)	3	7
	Gradual release from test position occurs	3−	6
	Moves through partial range of motion against gravity	2+	5
	Holds against slight pressure in test position	2+	4
	Movement through complete range of motion for the muscle being tested	2	3
	Movement through partial range of motion	2−	2
	Tendon becomes prominent or feeble contraction felt in muscle with no visible movement	1	1
	No contraction felt or seen in the muscle	0	0

**Table 2:** Distribution of study participants according to age (mean ± SD), BMI (median ± IQR), gender, lesion level, surgical status (NS—no surgery. S—surgery. IS—iterative surgery), and physical load (sedentary—S, medium physical activity—M, intense professional physical activity with overload—IP, intense physical activity with extended fixed position—IF and intense physical activity in the household—IG).

		Group 1	Group 2	Total	P
Age		59.19 ± 12.20	58.52 ± 12.08	58.85 ± 12.10	0.755 *
BMI		30.78 ± 5.49	29.41 ± 6.87	28.87 ± 6.43	0.566 **
Gender	Women	44	40	84	χ^2^ (1, *n* = 133) = 0.694, *p* = 0.405 ***
	Men	22	27	49	
Lesion level	Sacral	30	25	55	χ^2^ (1, *n* = 133) = 0.907, *p* = 0.341 ***
	Lumbar	36	42	78	
Surgical status	NS	30	34	64	χ^2^ (2, *n* = 133) = 0.552, *p* = 0.759 ***
	S	20	20	40	
	IS	16	13	29	
Physical load	S	19	11	30	χ^2^ (4, *n* = 133) = 15.42, *p* = 0.004 ***
	M	0	11	11	
	IP	12	6	18	
	IF	8	10	18	
	IG	27	29	56	

* Two sample *t*-test, ** Mann–Whitney U test, *** Chi-square test.

**Table 3:** The muscle strength scores obtained before the initiation of the treatment program (Before) and immediately upon its completion (After). The values are presented as Median ± Interquartile Range (IQR).

	Before	After	P (Wilcoxon Sign Rank Test)
Group 1	7.0 ± 1.0	10.0 ± 1.5	<0.001
Group 2	7.0 ± 1.0	8.0 ± 1.0	<0.001
P (Mann–Whitney U test)	0.008	<0.001	

**Table 4:** Difference between the muscle strength prior and after treatment (number of participants) categorized by surgical status: NS—no surgery, S—surgery, IS—iterative surgery.

Difference between the Muscle Strength Scores Prior and after the Treatment		NS	S	IS
Group 1	5 points	2	1	2
	4 points	14	5	9
	3 points	11	9	4
	2 points	3	5	1
	1 point	0	0	0
	0 points	0	0	0
Group 2	5 points	0	0	0
	4 points	0	0	0
	3 points	1	0	2
	2 points	5	4	3
	1 point	22	12	6
	0 points	6	4	2

**Table 5:** The ability to walk on tiptoes or heels scores obtained before the initiation of the treatment program (Before) and immediately upon its completion (After). Values are presented as Median ± Interquartile Range (IQR).

	Before	After	P (Wilcoxon Sign Rank Test)
Group 1	2.0 ± 0.75	0.0 ± 1.0	<0.001
Group 2	2.0 ± 1.0	1.0 ± 1.0	<0.001
P (Mann–Whitney U test)	0.867	<0.001	

**Table 6:** The median score of risk of falls parameter for both study groups obtained before the start of the treatment program (Before) and immediately after its completion (After). Values are presented as Median ± Interquartile Range (IQR).

	Before	After	P (Wilcoxon Sign Rank Test)
Group 1	2.0 ± 0.0	0.0 ± 1.0	<0.001
Group 2	2.0 ± 1.0	1.0 ± 1.0	<0.001
P (Mann–Whitney U test)	0.006	<0.001	

**Table 7:** Risk of falls score evolution based on surgical status: NS—no surgery, S—surgery, IS—iterative surgery.

Difference between the Risk of Falls Scores Prior and after the Treatment		NS	S	IS
Group 1	2 points	17	12	10
	1 point	13	8	6
	0 points	0	0	0
Group 2	2 points	0	0	1
	1 point	16	10	4
	0 points	18	10	8

## Data Availability

Data are available upon request.
